# Caregivers’ Experiences With a Web- and Mobile-Based Platform for Children With Medical Complexity and the Role of a Live Platform Coach: Thematic Analysis

**DOI:** 10.2196/43214

**Published:** 2023-07-05

**Authors:** Ainslie Claire Shouldice, Madison Beatty, Sherri Adams, Blossom Dharmaraj, Clara Moore, Jennifer Nan Stinson, Arti Desai, Leah Bartlett, Erin Culbert, Eyal Cohen, Julia Orkin

**Affiliations:** 1 Child Health Evaluative Sciences SickKids Research Institute The Hospital for Sick Children Toronto, ON Canada; 2 Division of Pediatric Medicine The Hospital for Sick Children Toronto, ON Canada; 3 Lawrence S Bloomberg Faculty of Nursing University of Toronto Toronto, ON Canada; 4 Department of Pediatrics University of Washington School of Medicine Seattle, WA United States; 5 Royal Victoria Regional Health Center Barrie, ON Canada; 6 Paediatric Services Credit Valley Hospital Mississauga, ON Canada; 7 Department of Pediatrics University of Toronto Toronto, ON Canada; 8 Institute of Health Policy, Management and Evaluation University of Toronto Toronto, ON Canada

**Keywords:** care coordination, care, children with medical complexity, children, chronic condition, electronic data, engagement, health information exchange, medical, patient care planning, pediatrics, usage, utilization

## Abstract

**Background:**

Children with medical complexity (CMC) are individuals with complex chronic conditions who have substantial health care needs, functional limitations, and significant use of health care. By nature of their health status, they have many care providers across multiple settings, making information sharing critical to their health and safety. Connecting2gether (C2), a web- and mobile-based patient-facing platform, was codeveloped with families to support and empower parental caregivers, improve information sharing, and facilitate care delivery. C2 also provided a live platform coach to conduct parental feedback and coaching sessions, which included answering questions, providing advice on usage, and addressing technological issues.

**Objective:**

This study was conducted to understand the experience of parental caregivers using the C2 platform and the role of the live platform coach. This study is a subset of a larger study assessing the feasibility of C2 in the care of CMC.

**Methods:**

Parental caregivers (n=33) participated in biweekly sessions to provide feedback and receive real-time platform use support from a trained research team member acting as a live platform coach. Parental caregivers were asked about the utility and usability of C2’s features. Questions, platform issues, and feedback were recorded on a standardized electronic data collection tool. A thematic analysis was performed to analyze parental comments, and codes were categorized into key themes. The number of comments corresponding with each code was quantified.

**Results:**

A total of 166 parental feedback and coaching sessions were conducted, with an average of 5 sessions per parental caregiver (range 1-7). There were 33 (85%) parental caregivers that participated in at least one coaching session. Technical issues and difficulties navigating C2 were addressed in real time during the sessions to encourage platform engagement. Four key themes were identified: (1) live platform coach, (2) barriers to platform usage and technical challenges, (3) platform requests and modifications, and (4) parent partnership and empowerment.

**Conclusions:**

Parental caregivers describe C2 as a valuable tool, acting as a facilitator for enhanced care coordination and communication. Parental caregiver feedback showed that the live platform coach was a critical tool in educating on platform use and addressing technological concerns. Further study of the use of the C2 platform and its role in the care of CMC is needed to understand the possible benefits and cost-effectiveness of this technology.

## Introduction

Children with medical complexity (CMC) are individuals with complex chronic conditions who have substantial health care needs, functional limitations, and significant use of health care [1]. CMC often require multiple care providers across various settings, including home, hospital, and school [2], and the unique challenges of navigating multiple levels of care often results in fragmented care [3]. Timely access to up-to-date medical information is critical in the care of CMC. However, this information is often challenging to obtain due to electronic medical record (EMR) access limitations and other communication barriers [3,4]. A significant burden is often placed upon parental caregivers to integrate information from various care providers and systems to address their child’s needs [5]. Streamlined communication, familial support, and health care team engagement are required to ensure that medical information is available to all members of a child’s care team [5].

The use of a shared web- and mobile-based patient-facing platform for CMC [4] may facilitate information sharing, communication, and coordination among parental caregivers and their child’s care team [6,7]. Previous research has explored the use of pediatric patient-facing platforms in order to improve patient information access, health outcomes, and communication with care providers [4]. Mobile health apps are positioned to deliver information outside of a clinical encounter [6]. Thus, mobile health apps can positively impact care outside of a health care setting. Studies of mobile health apps efficacy in pediatrics suggest that mobile health apps that involve usage by caregivers may have a more significant impact on health outcomes [8]. However, the use of mobile health apps in the care of CMC has been limited [6,8-11]. The complexity of this pediatric population, alongside previously identified caregiver burden [5], may benefit from a mobile health app with elevated levels of user support to enhance the caregiver experience and obtain maximal benefit from the app itself. Thus, the use of a live platform coach may be necessary to provide parental caregivers with the support required for platform use and accurate assessment of the use of these apps.

The overarching research question is to understand the parental caregiver experience of using Connecting2gether (C2), a web- and mobile-based patient-facing platform for parental caregivers of CMC. The objectives were to (1) identify barriers and challenges to platform uptake, (2) understand facilitators of parental use, and (3) evaluate the role of a live platform coach. This analysis is a substudy of a larger evaluation assessing the feasibility of the C2 platform in caring for CMC.

## Methods

### Connecting2gether Platform

C2 was developed to facilitate communication, care coordination, and information sharing between parental caregivers of CMC and their child’s care team using secure messaging, health tracking, educational resources, a web- and mobile-based schedule, care map [12], and shared care plans [13]. Parental caregivers could invite their child’s care team (ie, family members, health care providers, care coordinators, and teachers) to use C2 with them, hereinafter referred to as their “circle of care.” Invitations for care team members to join C2 were sent through the platform by parental caregivers upon providing a circle of care member email address. Upon receiving the invitation email, circle of care members were able to set up their profile in C2. Parental caregivers were able to control the ability of each circle of care member to view and edit their child’s profile. The live platform coach was able to assist parental caregivers in inviting care team members to the platform if they experienced difficulty with the invitation process. C2 was developed in partnership with a health technology solution company, NexJ Health, which provides evidence-based health solutions to those experiencing chronic conditions [14]. C2 was co-developed in partnership with parental caregivers and circle of care members of CMC. C2 was accessible through desktop, tablet, and mobile (iOS and Android) devices.

C2 also contained an automated points system where points were acquired when features were used (ie, inviting a care team member and sending a message) to enhance engagement.

### Live Platform Coach

The team clinical research coordinator (MB) was trained by NexJ Health for the role of live platform coach and was knowledgeable about the care of CMC. The live platform coach engaged in continued, weekly touchpoints with NexJ Health. The term “coaching” henceforth refers to the act of counseling and encouraging parental caregivers on platform use. The purpose of the live platform coach was to empower, support, and educate, building parental self-sufficiency in platform use. Unlike commonly used programmed chatbots or artificially intelligent and programmed helpers, entitled “virtual human assistants” [9], the live platform coach provided a more personalized approach to addressing parental concerns and needs related to the use of the C2 platform. The live platform coach facilitated feedback and coaching sessions with parental caregivers, acting in a role of mentorship and support, as well as gathering feedback comments. Feedback and coaching sessions were designed to collect information about parental caregivers’ perspectives and behaviors related to platform use and utility. The platform coach addressed user concerns, aided with platform navigation, provided suggestions on the use of platform features, and sent a weekly message to all parental caregivers through the secure messaging system. The messages were formulated based on parental caregiver feedback and included a welcome message, helpful platform tips, educational resources, and updates on platform features. Weekly messages were intended to increase parental caregiver engagement, address common usage issues, and bring attention to lesser-used platform features. The platform coach communicated regularly with parental caregivers using the secure messaging system and did not provide medical advice.

Parental caregivers who identified concerns in the web- and mobile-based form were contacted by the live platform coach. Issues were addressed in real time by the live platform coach and tracked throughout the study and were further addressed through regular platform updates by NexJ Health.

### Study Design and Population

Parental caregivers of CMC were recruited from the Complex Care Program (CCP) at The Hospital for Sick Children, Credit Valley Hospital, and Royal Victoria Regional Health Center in Ontario, Canada. To be eligible for the CCP, children must meet at least one criterion from each of the following conditions: technology dependence, users of high-intensity care, fragility, chronicity, and complexity [15].

Purposive sampling was used to ensure diversity in participant age, ethnicity, and geographical location. Nurse practitioners within the CCP were asked to draw upon their knowledge of parental caregiver experiences to recommend families for study participation. Primary caregivers of CMC who had been in the CCP for at least three months and had an active care plan were eligible for inclusion in the study. Parental caregivers were excluded if participation in the research study would be an added burden due to challenges including end of life, acute deterioration, hospitalization, parental physical or mental health concerns, or if caregivers did not speak English. English language proficiency was necessary for platform navigation and the use of the educational materials.

Study information was sent by post to eligible parental caregivers. Recruitment was conducted by phone and informed consent was obtained. Parental caregivers were recruited between July and November 2019 and used the C2 platform for 6 months.

### Data Collection

Parental caregivers completed a demographics questionnaire at the beginning of the study in person or through REDCap (Research Electronic Data Capture), a web- and mobile-based electronic survey tool [16]. Chart review was conducted by a research team member for participating CMC to obtain additional demographic information regarding the clinical characteristic of the patient sample.

Parental caregivers were asked to participate in biweekly feedback and coaching sessions for the first month and in monthly sessions thereafter. Parental caregivers were given a choice at the study onset of completing the sessions through the phone, in person, or through REDCap, which contained standardized questions with open-ended responses (Multimedia Appendix 1). Parental caregivers were asked about their experience using C2, its usefulness, and each main platform feature such as “If not using any trackers, is there a reason you have not been using the trackers? Is there anything I can help you with?” The web- and mobile-based form was sent at predetermined time points and automatic reminders were sent if the form was not completed within a week. Feedback was collected verbatim for parental caregivers who completed the web- and mobile-based sessions. Telephone and in-person sessions were summarized by the live platform coach through the use of typed notes. All feedback and coaching session data were collected and managed using REDCap.

Participants received a CAD $20 and CAD $40 gift card (a currency exchange rate of CAD $1=US $0.74 is applicable) after completing baseline questionnaires and at the end of the study, respectively. Parental caregivers also received a CAD $5 gift card when they reached point milestones on C2 (1000, 2500, 5000, and 10,000 points).

### Data Analysis

Demographic and chart review data were analyzed using descriptive statistics to contextualize the parental caregivers and CMC in the study. Responses from the feedback and coaching sessions were collected and organized based on the interview questions targeted to platform features.

Data from the sessions were analyzed using thematic analysis methods proposed by Braun and Clarke [17] by 3 members of the research team (JO, ACS, and MB). Thematic analysis is flexible, allowing for the production of a robust, nuanced, and detailed account of complex data. Thematic analysis has been successfully used in the analysis of open-ended interview question responses [18-20]. In this study, thematic analysis was chosen as this methodology is able to encompass the breadth of parental caregiver experiences in a complex and diverse population.

In this study, thematic analysis was conducted in alignment with the 6 stages for thematic analysis proposed by Braun and Clarke [17]. In stage 1 of the framework, data were first reviewed to promote familiarization (ACS). In stage 2, codes and subcodes were systematically developed across the entire dataset to categorize feedback based on platform features and parental caregiver experience (ACS). Codes were reviewed by other research team members (JO and MB) to reduce bias. Codes and subcodes were merged and refined to better reflect parental caregiver feedback, and a code tree was synthesized (ACS, JO, and MB). In stage 3, codes were collated into themes. Themes were developed inductively to describe the parental caregiver experience with C2 (ACS). In stage 4, themes were reviewed and amended by all 3 coding members of the research team to reflect the comments provided (ACS, JO, and MB). In accordance with stage 5, ongoing analysis of data was used to develop specific definitions and names for each theme. In stage 6, specific representative examples from the data were chosen to aid in communication of each theme.

The data were analyzed quantitatively to determine the frequency of parental caregiver comments based on each subcode and the number of parental caregivers who provided feedback under each code. The number of sessions completed by each parental caregiver were quantitatively determined.

### Ethics Approval

Institutional research ethics approval was obtained at Credit Valley Hospital (REB #973), The Hospital for Sick Children (SickKids, REB1000060804), and Royal Victoria Regional Health Centre (REB #R18-013) in Ontario, Canada. Informed consent was obtained from all participants. Research was conducted in accordance with all applicable guidelines and regulations. Study data were deidentified prior to data analysis. A username and password were required to access C2. C2 is a secure, cloud-based platform. All C2 data were stored on IBM Softlayer servers, which are HIPAA, FedRAMP, PCI, and FISMA certified and fully compliant with ISO 27001, 27017, 27018. All stored data were encrypted and transferred within the system using only secure HTTPS connections.

## Results

### Platform Usage

**Figure 1:**
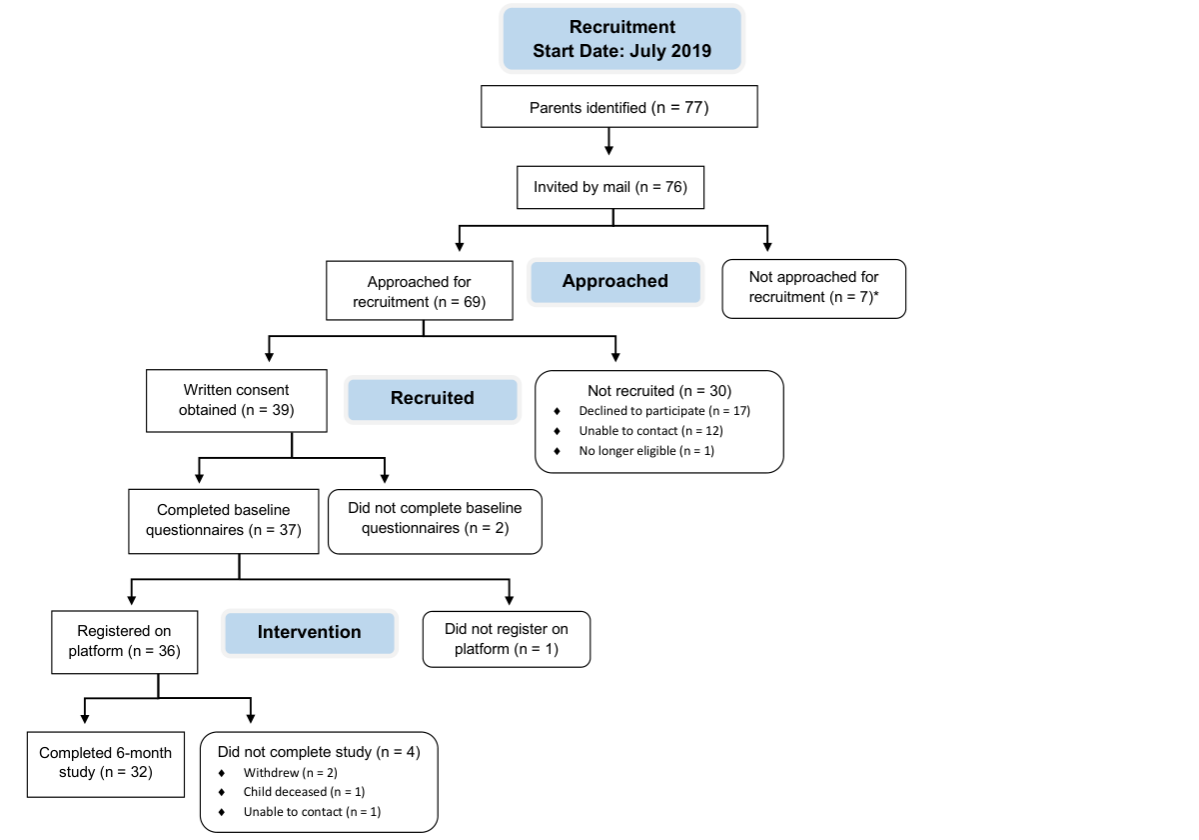
Flow diagram of parental caregiver recruitment. *These participants were not approached because recruitment had closed.

A total of 39 parental caregivers consented to participate in the research study, and 37 parental caregivers completed the baseline data questionnaires (Figure 1). Out of these, 36 parental caregivers registered on the platform and 33 parental caregivers participated in at least 1 coaching session, with an average of 5 sessions per parental caregiver (n=33, range 1-7). Of the 3 parental caregivers who did not complete any feedback and coaching sessions, 2 withdrew from the study before completing a session, and 1 could not be contacted. Out of 252 possible parental feedback and coaching sessions, 166 were completed,demonstrating high parental engagement. A total of 42 (25%) feedback and coaching sessions occurred over the phone (range 5-15 minutes in length) and 122 (73%) feedback sessions were completed on the web. A total of 2 (0.01%) feedback sessions occurred in person. CMC and parental caregiver characteristics are included in Tables 1 and 2, respectively.

**Table 1:** Demographic characteristics of children with medical complexity (CMC) (n=37).

Characteristic		Children with medical complexity
**Sex, n (%)**		
	Female	14 (38)
	Male	23 (62)
**Length of time in complex care program, n (%)**		
	<1 year	3 (8)
	1-3 years	9 (24)
	>3 years	25 (68)
**Number of diagnoses, n (%)**		
	1-5	2 (5)
	6-10	19 (51)
	11-15	15 (40)
	>15	1 (3)
**Number of medications, n (%)**		
	1-10	15 (41)
	11-20	19 (51)
	21-30	3 (8)
**Number of technologies, n (%)**		
	<5	11 (30)
	5-10	19 (51)
	11-15	7 (19)
**Technology devices^a^, n**		
	Feeding technology^b^	45
	Respiratory technology^c^	67
	Vascular access device^d^	4
	Mobility device^e^	111
	Communication device^f^	4
	Other invasive technology^g^	1
**Number of subspecialists^h^, n (%)**		
	<5	1 (3)
	5-10	18 (49)
	11-15	18 (49)

^a^Each CMC used multiple technologies.

^b^Feeding technology: devices to obtain nutrition (ie, G-J tube, feeding pump).

^c^Respiratory technology: devices to assist with and monitor respiratory function (ie, tracheostomy, nebulizer, oxygen, heated high flow, bilevel positive airway pressure cough assist, oximeter).

^d^Vascular access device: devices for venous access (ie, central venous line and peripherally inserted central catheter).

^e^Mobility device: devices to improve personal mobility and movement (ie, special stroller, specialized wheelchair, walker, stander, portable Hoyer lift, bath chair, tomato chair, shoe lift, and ankle and foot orthotics).

^f^Communication device: devices used for communication purposes (ie, communication systems, cochlear implants, GO Talk board, iPad, and writing aids).

^g^Other invasive technology includes vagal nerve stimulator.

^h^Most used specialist included: neurology, respirology, cardiology, genetics, and hematology.

**Table 2:** Demographic characteristics of parental caregivers (n=37).

Characteristics		Parental caregivers, n (%)
**Role**		
	Mother	30 (81)
	Father	7 (19)
**Age (years)**		
	20-40	19 (51)
	>40	16 (43)
	Did not answer	2 (6)
**Primary language**		
	English	34 (92)
	Other	3 (8)
**Ethnic background**		
	Caribbean	3 (8)
	East and South Asian	3 (8)
	European ancestry	25 (67)
	Latin, Central and South American	1 (3)
	Other	2 (6)
	Prefer not to answer	3 (8)
**Marital status**		
	Single or divorced	4 (12)
	Married or common law	30 (80)
	Prefer not to answer	3 (8)
**Education**		
	High school	2 (5)
	Postsecondary school	21 (57)
	Professional or graduate degree	13 (35)
	Prefer not to answer	1 (3)
**Employment status**		
	Employed full-time or part-time	19 (51)
	Primary caregiver	12 (32)
	Unemployed	3 (8)
	Other	2 (5)
	Prefer not to answer	1 (3)
**Annual household income (CAD $^a^)**		
	0-59,999	9 (24)
	60,000-89,999	6 (16)
	>90,000	18 (49)
	Prefer not to answer	4 (11)

^a^A currency exchange rate of CAD $1=US $0.74 is applicable.

### Quantitative Analysis of Functionality and Features

Functionality describes the overall ease of use and visual presentation of the platform. Nonparticipation refers to a lack of platform use and participation by members of the circle of care, including healthcare providers, educators, and parents. Parental caregivers’ comments most commonly referred to C2’s features (ie, educational resources and secure messaging), accounting for 65.3% (335/513) of the total comments (Table 3). Of these features, the shared care plans and secure messaging were most frequently discussed (103/513, 20.1% and 82/513, 16%, respectively; Table 3). Parental caregivers provided the fewest comments on platform functionality (2/513, 0.4%) and the schedule feature (8/513, 1.6%; Table 3).

**Table 3:** Number of items of feedback from parental caregivers by code.

Code	Items of feedback (n=513), n (%)^a^	Parental caregivers who commented (n=33), n (%)^b^
Care plan	103 (20.1)	31 (93.9)
Secure messaging	82 (16)	32 (97)
Health trackers	67 (13)	31 (93.9)
Nonparticipation	63 (12.3)	31 (93.9)
Health library and workbooks	41(8)	26 (78.8)
Other feedback^c^	38 (7.4)	21 (63.6)
Care map	34 (6.6)	25 (75.8)
Live platform coach	32 (6.2)	32 (97)
Platform invitations	24 (4.7)	24 (72.7)
Technical issues	12 (2.3)	12 (36.4)
Schedule	8 (1.6)	8 (24.2)
Questions about research study	7 (1.4)	7 (21.2)
Functionality	2 (0.4)	2 (6.1)

^a^A piece of feedback is defined as a comment from a parental caregiver related to a specific aspect of the platform.

^b^Parental caregivers who provided comments falling under multiple subcodes were counted for each subcode.

^c^Other feedback includes feedback related to concerns about security, learning curve, integration, and positive comments about platform interface and usability

### Thematic Summary of Parental Caregiver Feedback

**Figure 2:**
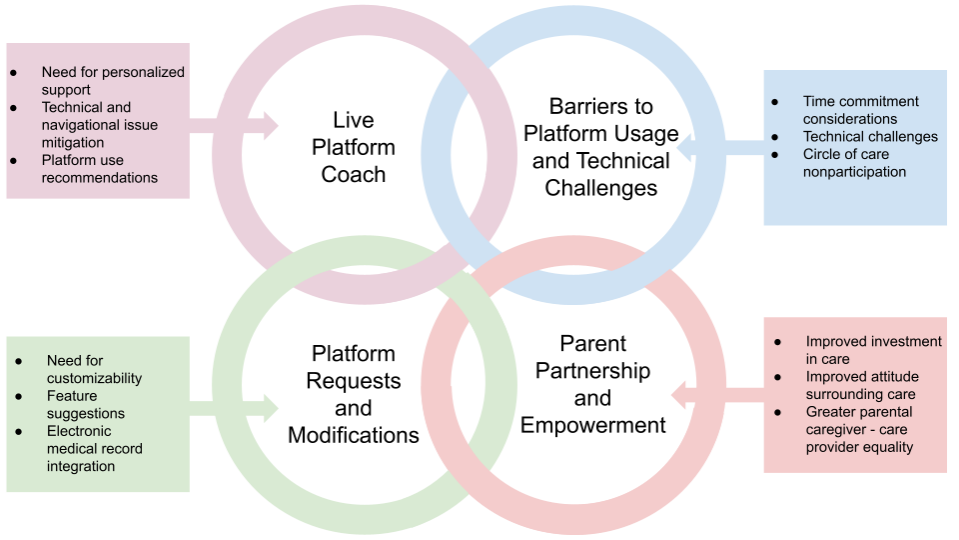
Themes describing the parental caregiver experience of using Connecting2gether.

To address the 3 study objectives, parental caregiver feedback was analyzed to identify key themes describing the parental caregiver experience using C2. From the analysis, four key themes emerged: (1) live platform coach, (2) barriers to platform usage and technical challenges, (3) platform requests and modifications, and (4) parental caregiver partnership and empowerment (Table 4 and Figure 2).

**Table 4:** Demonstration of platform usage and parental feedback relating to each identified theme.

Theme	Parental feedback
Live platform coach	“I messaged [platform coach] on platform to get help editing a wellness tracker entry.” (P16) “[Platform coach] changed [the weight tracker to kilogram] for [me] and told [me] how to locate units under settings.” (P22)
Barriers to platform usage and technical challenges	“I asked [my] child’s school nurses to join in the care circle. They were reluctant to as for their agent’s policy.” (P14) “There were parts of the program I wanted to use such as care maps. They just didn’t work.” (P1) “This past week has been rough with child, [I] haven’t had as much time to go through the platform.” (P4)
Platform requests and modifications	“[I had] trouble adding signs and symptoms tracker–[I] wanted to create own rather than adding from the list.” (P16) “It would be useful to have home care nurses charting on the platform to be able to share with health care providers and nurse practitioners.” (P14)
Parental caregiver partnership and empowerment	“[I] had a meeting with school–printed off the care map and care plan. [I] felt more confident sitting in the meeting with the documents.” (P41) “[Connecting2gether] opened [my] eyes to the possibility to negotiate dialogue with health care team. [I felt] empowered to participate in child's care.” (P8)

### Thematic Analysis

#### Live Platform Coach

Parental caregivers described the emerging role of the live platform coach as a key facilitator of platform use. The role of the live platform coach evolved into assisting with platform navigation, providing suggestions on the use of platform features, addressing technical errors, and gathering parental feedback comments. The live platform coach was able to work with parental caregivers to aid in password retrieval, unit conversions, and technical issue mitigation, potentially enhancing parental engagement. For example, the platform coach helped navigate between metric and imperial conversions for the health trackers, saving parental caregivers time and maintaining a positive experience. Parental caregivers also often asked about how to use specific platform features (ie, care map and care plans). The platform coach was able to provide suggestions as to how the platform could support their individual needs. For example, the platform coach provided suggestions on the use of the care map, such as the type of information that may be useful to add to a care map and who to share it with. The platform coach was also available to field parental caregiver questions and support platform use. A total of 97% (32/33) of parental caregivers commented on their use of the live platform coach, highlighting the importance of this role and parental caregiver support. The majority of these comments described the use of the platform coach in navigating platform use, mitigating technical issues, and suggesting avenues of platform use.

#### Barriers to Platform Usage and Technical Challenges

Parental caregiver feedback on barriers to platform usage included challenges such as the time commitment to use the platform, circle of care nonparticipation, lack of familiarity with the platform, and a lack of platform usefulness during periods of health stability (Table 3). Of parental caregivers, 24.2% (8/33) reported that platform set-up was a time-consuming process as some of the child’s health information had to be manually entered, leading to decreased participation. Lack of engagement of circle of care members was a common barrier reported by parental caregivers and was usually due to organizational policies or lack of familiarity with the platform (Table 3).

Parental caregivers described technical challenges as a significant barrier to platform use. Common technical challenges included the mobile app crashing and a lengthy website loading time. Though only 36.4% (12/33) of parental caregivers discussed technical challenges (Table 3), the parental caregivers who experienced these challenges reported that this had a significant impact on their use of C2. Technical issues were mitigated through contact with the platform developer by the platform coach and the use of periodic software updates. The platform coach advised parental caregivers on simple technical solutions such as updating the app or device software to mitigate some of the technical issues.

#### Platform Requests and Modifications

Many parental caregivers requested increased customizability on certain aspects of the platform features, such as the secure messaging or shared care plans, and suggested new platform functions, particularly related to the health trackers and the schedule feature. For example, several parental caregivers suggested adding a feature allowing for gaps in sleep to be tracked to reflect the total hours of sleep more accurately. Parental caregivers also requested the ability to sync the web- and mobile-based C2 schedule with their own personal calendars. Parental caregivers also felt that integration of C2 with the hospital’s patient-facing EMR would enhance usability and indicated that the lack of integration negatively impacted their use of C2 as it required multiple platforms to be used for a comprehensive understanding of their child’s health care needs.

#### Parental Caregiver Partnership and Empowerment

A key facilitator of parental caregiver platform use was the feeling of renewed parental caregiver-provider partnership and empowerment. Though feedback and coaching session questions focused on discerning methods of platform use and mitigating issues, roughly one-third of parental caregivers reported feelings of partnership with the medical team and empowerment through an improvement in attitude and investment in their child’s care after using C2. Parental caregivers felt more involved in their child’s care and as though they were acknowledged by their child’s care providers. For example, one caregiver reported that they had felt that the health care team provided information and they received information prior to the study start. They stated that the platform helped to change and equalize that dynamic. Many parental caregivers appreciated the ease of communication with other members of their child’s care team. Some parental caregivers indicated interest in C2 being available for a longer duration and to other families of CMC.

## Discussion

### Principal Results

To our knowledge, this is one of the first studies to assess the feasibility of a web- and mobile-based, patient-facing platform for parental caregivers of CMC. Upon examination of parental feedback data, the parental caregiver perspective on the use of CMC was characterized and organized into four key themes: (1) barriers to platform usage and technical challenges, (2) platform requests and modifications, (3) parental caregiver partnership and empowerment, and (4) live platform coach.

### Parental Caregiver Partnership and Empowerment

Some parental caregivers reported renewed feelings of partnership with their child’s care team members and a greater ability to participate in their child’s care. Parental caregivers of CMC face challenges associated with fragmented care that impacts their partnership with health care providers [4]. This finding replicates previous study findings, where parental caregivers reported that they felt their expertise in their child’s care was not acknowledged or valued by members of the medical team [4]. Previous studies have identified a need for family-centered care (FCC), where health care decisions are made based on a partnership between health care providers and families [4,21]. A primary challenge in the implementation of FCC is a lack of communication and collaboration. The outcome of improved parental caregiver empowerment following the use of C2 demonstrates that the use of a web- and mobile-based platform with a live platform coach can be considered as a facilitator of enhanced FCC.

### Live Platform Coach

Parental caregivers reported that technical challenges also impacted their use of C2. Previous research on mobile health apps indicates technical challenges, including learnability, system design, and interoperability, affect platform usage [22]. These issues were mitigated in part by using the live platform coach, who provided suggestions to circumvent technical issues. The instrumental nature of the live platform coach to platform use was an unanticipated outcome of this study, thus feedback session questions did not directly target its use. The volume of parental caregiver comments describing the use of the live platform coach indicates the critical nature of this role.

The parental caregiver experience suggests that C2 was a useful tool for parental caregivers in the care of CMC. Though barriers existed, they were partially mitigated by the role of the live platform coach. CMC are a diverse population with a wide variety of needs, thus a human in the role of a platform coach is required to provide a flexible and tailored response to web- and mobile-based patient-facing platforms supporting the needs of parental caregivers of this population. Though the use of virtual human assistants has been described in many web- and mobile-based health platforms in adult populations, the role of a live platform coach has not been studied in pediatric populations. Support implemented in previous web-based health care platforms typically takes the form of an artificially intelligent virtual human assistant, as opposed to a person engaged in the role of platform support and partnership [9]. Unlike the platform coach role used in C2, the role of a virtual human assistant describes programmable features, like chatbots, that have been implemented in some mobile health apps for adult populations [9]. In analyses of the use of a virtual human assistant, more “human-like” characteristics of the avatar used and the programmed response including empathy and self-disclosure result in more positive parental caregiver experiences. A live platform coach may be beneficial over a virtual human assistant, as the live platform coach can provide a greater degree of personalization and specialization necessary for this population where feasible. The results of this study suggest that the live platform coach is a critical component of C2, and that widespread implementation of the platform would require an individual to function in a similar role for improved platform uptake.

### Parental Caregiver Use and Platform Integration

Parental caregiver feedback suggests that C2 is a useful tool to have, when necessary, but its daily use is not always feasible or helpful. Caring for CMC is a significant time commitment [5], and a lack of time was reported by parental caregivers to be one of the most significant barriers to platform use (Table 3). Previous studies of adults using mobile health-related interventions identified that health issues often prevented patients from participating due to limitations imposed by the time required to manage their condition or pain, for example [23]. Similarly, several parental caregivers in the study reported decreased platform use due to a lack of time secondary to intensive care provision for their medically complex child. Thus, this study found that lack of time often perturbed the use of C2 from fitting parental caregivers’ daily schedules. Parental caregivers found that the platform was most helpful when it could be used as a tool to track health patterns over time as needed instead of being used daily, particularly in establishing timelines during consultation with physicians and other health care professionals.

Previous studies on mobile health platforms showed that a lack of integration with EMR was a barrier as the use of other platforms or EMR themselves was prioritized due to convenience [24]. As reported by parental caregivers in the study, parental caregivers found that C2 was a valuable tool, but that further integration with existing EMR was necessary to improve usefulness. The C2 platform was not embedded within the hospital’s EMR; thus, providers and parental caregivers had to manually input changes and log into a separate system. Parental caregivers stated that the integration of MyChart’s schedule or results reporting tools would have improved platform usefulness. Due to the nature of the feasibility study, EMR integration was not addressed. Platform efficacy must be determined prior to EMR integration being considered, and a large-scale assessment of feasibility must be performed. Partnership with parental caregivers and a user-centered design approach is crucial to ensure that the future iteration of a web- and mobile-based patient-facing platform for CMC is tailored to parental needs, including increased customizability.

### Comparison With Previous Work

Previous studies have identified multiple challenges in care coordination for CMC [4]. The unique complexity of this population requires the investigation of unique solutions. In partnership with NexJ Health, the existing NexJ Connected Wellness (NCW) was adapted to create C2 [14]. Previous studies evaluating the use of NCW have been conducted on adult populations [25]. To adapt to the unique CMC pediatric population, features from NCW including workbooks and health trackers were tailored to the CMC population, providing increased platform adaptability, layout, and functionality. Previous studies on care coordination challenges in CMC identified that the implementation of shared systems that can be accessed by all members of a child’s care team in real time may help mitigate these challenges [4]. This study expands upon previous work to evaluate parental caregiver perspectives on a novel technological solution to improve care coordination and information-sharing.

### Limitations

Given parental caregiver preference to complete sessions remotely or by phone, data recording methodology for feedback and coaching sessions varied. Summarization and verbatim transcription were both used. The use of summarization is a limitation of this study as it may have resulted in interviewer bias. Further study limitations involve study exclusion criteria. The study population excluded individuals that did not speak English, and future research is needed to understand the feasibility of C2 for families who prefer languages other than English. Further, families involved in the study were approached based on a nurse practitioner’s evaluation that they would be able to use the technology and provide constructive feedback, and that it would be appropriate for families to participate, which may have also introduced bias. Additionally, the majority of parental caregivers were of European ancestry and had completed a postsecondary degree, with close to half of participants having an annual household income of more than CAD $90,000. As a result, the breadth of needs of parental caregivers belonging to lower socioeconomic status groups or those from historically marginalized groups may not have been encompassed by the study.

### Conclusions

It is well understood that caring for CMC involves communicating with multiple health care providers across a variety of settings and a significant amount of care coordination by parental caregivers, amounting to a large time commitment for families. The C2 platform was described as a useful tool by parental caregivers to enhance communication and information access in the care of CMC. Improved access to information and educational resources, alleviation of caregiver burden, and tracking of health trends may lead to improved health outcomes for this population. The live platform coach facilitated platform use, mitigated technical issues, and enhanced the experience of parental caregivers with C2. Future patient-facing platforms for CMC should consider the use of a live platform coach to provide personalized platform support and improve platform utility.

## Appendix

Multimedia Appendix 1 Parental caregiver online feedback and coaching session questionnaire.

## Abbreviations

C2: Connecting2gether
CCP: Complex Care Program
CMC: children with medical complexity
EMR: electronic medical record
FCC: family-centered care
NCW: NexJ Connected Wellness

## References

[1] Cohen E, Kuo DZ, Agrawal R, Berry JG, Bhagat SK, Simon TD, Srivastava R (2011). Children with medical complexity: an emerging population for clinical and research initiatives. Pediatrics.

[2] Kuo DZ, Melguizo-Castro M, Goudie A, Nick TG, Robbins JM, Casey PH (2015). Variation in child health care utilization by medical complexity. Matern Child Health J.

[3] Mosquera RA, Avritscher EBC, Pedroza C, Bell CS, Samuels CL, Harris TS, Eapen JC, Yadav A, Poe M, Parlar-Chun RL, Berry J, Tyson JE (2021). Hospital consultation from outpatient clinicians for medically complex children: a randomized clinical trial. JAMA Pediatr.

[4] Adams S, Beatty M, Moore C, Desai A, Bartlett L, Culbert E, Cohen E, Stinson J, Orkin J (2021). Perspectives on team communication challenges in caring for children with medical complexity. BMC Health Serv Res.

[5] Kuo DZ, McAllister JW, Rossignol L, Turchi RM, Stille CJ (2018). Care coordination for children with medical complexity: whose care is it, anyway?. Pediatrics.

[6] Wang G, Wignall J, Kinard D, Singh V, Foster C, Adams S, Pratt W, Desai AD (2021). An implementation model for managing cloud-based longitudinal care plans for children with medical complexity. J Am Med Inform Assoc.

[7] Desai AD, Wang G, Wignall J, Kinard D, Singh V, Adams S, Pratt W (2020). User-centered design of a longitudinal care plan for children with medical complexity. J Am Med Inform Assoc.

[8] Fedele DA, Cushing CC, Fritz A, Amaro CM, Ortega A (2017). Mobile health interventions for improving health outcomes in youth: a meta-analysis. JAMA Pediatr.

[9] Zhang L, Babu SV, Jindal M, Williams JE, Gimbel RW (2019). A patient-centered mobile phone app (iHeartU) with a virtual human assistant for self-management of heart failure: protocol for a usability assessment study. JMIR Res Protoc.

[10] Genes N, Violante S, Cetrangol C, Rogers L, Schadt EE, Chan YY (2018). From smartphone to EHR: a case report on integrating patient-generated health data. NPJ Digit Med.

[11] Mirkovic J, Kaufman DR, Ruland CM (2014). Supporting cancer patients in illness management: usability evaluation of a mobile app. JMIR Mhealth Uhealth.

[12] Adams S, Nicholas D, Mahant S, Weiser N, Kanani R, Boydell K, Cohen E (2017). Care maps for children with medical complexity. Dev Med Child Neurol.

[13] Adams S, Cohen E, Mahant S, Friedman JN, Macculloch R, Nicholas DB (2013). Exploring the usefulness of comprehensive care plans for children with medical complexity (CMC): a qualitative study. BMC Pediatr.

[14] NexJ Health-Advanced Virtual Care.

[15] (2023). Complex Care for Kids in Ontario. Provincial Council for Maternal and Child Health.

[16] Harris PA, Taylor R, Minor BL, Elliott V, Fernandez M, O'Neal L, McLeod L, Delacqua G, Delacqua F, Kirby J, Duda SN, Redcap Consortium (2019). The REDCap consortium: building an international community of software platform partners. J Biomed Inform.

[17] Braun V, Clarke V (2006). Using thematic analysis in psychology. Qualitative Res Psychol.

[18] Stewart D, Burrow H, Duckworth A, Dhillon J, Fife S, Kelly S, Marsh-Picksley S, Massey E, O'Sullivan J, Qureshi M, Wright S, Bowers L (2015). Thematic analysis of psychiatric patients' perceptions of nursing staff. Int J Ment Health Nurs.

[19] Beng TS, Guan NC, Seang LK, Pathmawathi S, Ming MF, Jane LE, Chin LE, Loong LC (2014). The experiences of suffering of palliative care patients in Malaysia: a thematic analysis. Am J Hosp Palliat Care.

[20] Molin KR, Egerod I, Valentiner LS, Lange P, Langberg H (2016). General practitioners' perceptions of COPD treatment: thematic analysis of qualitative interviews. Int J Chron Obstruct Pulmon Dis.

[21] Kuo DZ, Houtrow AJ, Arango P, Kuhlthau KA, Simmons JM, Neff JM (2012). Family-centered care: current applications and future directions in pediatric health care. Matern Child Health J.

[22] Gurupur VP, Wan TTH (2017). Challenges in implementing mHealth interventions: a technical perspective. Mhealth.

[23] Nielsen AS, Kidholm K, Kayser L (2020). Patients' reasons for non-use of digital patient-reported outcome concepts: a scoping review. Health Informatics J.

[24] Logan AG (2013). Transforming hypertension management using mobile health technology for telemonitoring and self-care support. Can J Cardiol.

[25] Wayne N, Perez DF, Kaplan DM, Ritvo P (2015). Health coaching reduces HbA1c in type 2 diabetic patients from a lower-socioeconomic status community: a randomized controlled trial. J Med Internet Res.

