# Supplementary material for: Caregivers’ Experiences With a Web- and Mobile-Based Platform for Children With Medical Complexity and the Role of a Live Platform Coach: Thematic Analysis
Source: JMIR Pediatr Parent. 2023 Jul 5;6:e43214. doi: 10.2196/43214 (PMC10357368; doi:10.2196/43214)

*Confidential*

**Parent Check-in**

*Page 1*

Please complete the survey below.

Thank you!

Check-in number

__________________________________

Date of last check-in

__________________________________

Date of completion

__________________________________

**ISSUES/QUESTIONS/FEEDBACK**

Have you had any issues with the platform since your

last check-in?

__________________________________________

Do you have any questions about anything on the

platform?

__________________________________________

Do you have any feedback about the platform that you

want to share?

__________________________________________

**MEDICAL CARE PLAN**

| Has your child had any diet or medication changes | Yes - Diet |
| --- | --- |
| since your last check-in? | Yes - Medication |
|  | No |
|  |  |
| Did you update the diet or medication sections of the | Yes - Diet |
| medical care plan on Connecting2gether? | Yes - Medication |
|  | No |
|  |  |
| What were the changes? |  |
|  | __________________________________________ |
|  |  |
| Is there a reason you have not updated the C2 care |  |
| plan? | __________________________________________ |
|  |
|  |  |
| Have you made any changes to the medical care plan | Yes |
| since your last check-in? | No |
|  |  |
| Please describe the changes (i.e. field added, items |  |
| changed): | __________________________________________ |
|  |
|  |  |
| Is there anything I can help you with? |  |
|  | __________________________________________ |


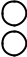


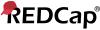


*Confidential*

*Page 2*

Have you used the medical care plan since your last check-in?

Yes


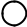


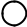
 No

How have you used the medical care plan?

__________________________________________

Is there a reason why not?

__________________________________________

**SCHOOL CARE PLAN**

Have you created a school care plan?

Yes


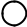


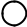
 No

Is there a reason why you have not created a school

care plan? Is there anything I can help you with?

__________________________________________

Have you made any changes to the school care plan since your last check-in?

Yes


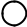


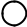
 No

Please describe the changes (i.e. fields added, items

changed):

__________________________________________

Is there anything I can help you with?

__________________________________________

Have you used the school care plan since your last check-in?

Yes


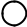


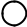
 No

How have you used the school care plan?

__________________________________________

Is there a reason why you have not used the school

care plan?

__________________________________________

**HOME CARE PLAN**

Have you created a home care plan?

Yes


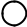


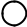
 No

Is there a reason why you have not created a home care

plan? Is there anything I can help you with?

__________________________________________

Have you made any changes to the home care plan since your last check-in?

Yes


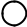


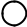
 No


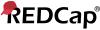


*Confidential*

*Page 3*

Please describe the changes (i.e. fields added, items

changed):

|  | __________________________________________ |
| --- | --- |
|  |  |
| Is there anything I can help you with? |  |
|  | __________________________________________ |
|  |  |
| Have you used the home care plan since your last | Yes |
| check-in? | No |
|  |  |
| How have you used the home care plan? |  |
|  | __________________________________________ |
|  |  |
| Is there a reason you have not used the home care |  |
| plan? | __________________________________________ |
|  |


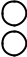


**TRACKERS**

Which trackers are you currently using?

|  | __________________________________________ |
| --- | --- |
|  |  |
| If not using any trackers, is there a reason you have |  |
| not been using the trackers? Is there anything I can | __________________________________________ |
| help you with? |

**EDUCATIONAL CONTENT**

| Have you used any educational content since your last | Yes |
| --- | --- |
| check-in (i.e. workbooks or health library)? | No |
|  |  |
| If no, is there a reason why you have not used this |  |
| feature? Is there anything I can do to help? | __________________________________________ |
|  |


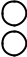


**CARE MAPS**

| Have you created a care map? | Yes |
| --- | --- |
|  | No |
|  |  |
| Is there a reason why you have not created a care map? |  |
| Is there anything I can do to help you with the care | __________________________________________ |
| map? |
|  |  |
| Have you updated the care map since your last | Yes |
| check-in? | No |
|  |  |
| Please describe the changes (i.e. bubble added, items |  |
| changed, etc.) | __________________________________________ |
|  |


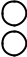

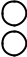


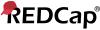


*Confidential*

*Page 4*

Is there a reason why you have not updated the care

map? Is there anything that I could help you with?

|  | __________________________________________ |
| --- | --- |
|  |  |
| Have you used the care map since your last check-in? | Yes |
|  | No |
|  |  |
| How have you used the care map? |  |
|  | __________________________________________ |
|  |  |
| Is there a reason why you have not used the care map? |  |
| Is there anything that I could help you with? | __________________________________________ |
|  |


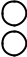


**MESSAGING**

| Have you been using the secure messaging feature? | Yes |
| --- | --- |
|  | No |
|  |  |
| What were the main reasons for sending the messages? |  |
|  | __________________________________________ |
|  |  |
| Is there a reason why you have not been using the |  |
| secure messaging feature? Is there anything that I can | __________________________________________ |
| do to help you with that feature? |
|  |  |
| Have you been contacting your health care team through | Yes |
| C2 instead of email? | No |
|  |  |
| Is there a reason why not? |  |
|  | __________________________________________ |


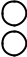

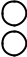


**CIRCLE OF CARE**

| Have you added anyone to your circle of care since | Yes |
| --- | --- |
| your last check-in? | No |
|  |  |
| Please describe the relationship: |  |
|  | __________________________________________ |
|  |  |
| Is there a reason why you have not invited anyone to |  |
| your circle of care? Is there anything I can help you | __________________________________________ |
| with? |


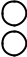


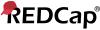

Supplement: Multimedia Appendix 1 [file pediatrics_v6i1e43214_app1.doc]
